# Structural Barriers to HIV Prevention and Services: Perspectives of African American Women in Low-Income Communities

**DOI:** 10.1177/10901981221109138

**Published:** 2022-07-20

**Authors:** Shelby Rimmler, Carol Golin, James Coleman, Hayley Welgus, Sarah Shaughnessy, Leah Taraskiewicz, Alexandra F. Lightfoot, Schenita D. Randolph, Linda Riggins

**Affiliations:** 1 The University of North Carolina at Chapel Hill, Chapel Hill, NC, USA; 2 Gillings School of Global Public Health, Chapel Hill, NC, USA; 3 North Carolina Institute of Medicine, Chapel Hill, NC, USA; 4 Gender and Development Consultant, Chiang Mai, Thailand; 5 City of Raleigh Planning & Development, Raleigh, NC, USA; 6 Duke University School of Nursing, Durham, NC, USA

**Keywords:** HIV prevention, sexual health, African American women, structural determinants of health

## Abstract

**Background:**

African American women are at a disproportionate HIV risk compared with other U.S. women. Studies show that complex structural and social determinants, rather than individual behaviors, place African American women at greater risk of HIV infection; however, little is known about women’s views of what puts them at risk.

**Aims:**

This study sought to comprehend the perceptions of African American women living in low-income housing regarding the factors that influence both their personal sexual health behaviors and use of HIV prevention services.

**Methods:**

We conducted seven focus groups with 48 African American women from 10 public housing communities in a small city in the southeastern United States. We analyzed the focus group transcripts using thematic data analysis to identify salient themes and points of interest related to the study aim.

**Results:**

Women identified factors related to the health care system (trustworthiness of the health care system), the external environment (racism, classism, patriarchal structures, and violence/crime), as well as predisposing (health beliefs, stigma, and gender norms), enabling (agency to negotiate gendered power), and need (perceived HIV risk and perceptions of partner characteristics) features of individuals in the population.

**Conclusion:**

African American women living in public housing are especially vulnerable to HIV infection due to intersectional discrimination based on racism, classism, gender power dynamics, and community conditions. Our findings confirm the need to develop HIV intervention programming addressing intersectional identities of those making up the communities they plan to address, and being informed by those living in the communities they plan to act on.

The southeastern region of the United States is disproportionately affected by the human immunodeficiency virus (HIV) epidemic, with twice as many new diagnoses as other U.S. regions (Centers for Disease Control and Prevention [CDC], 2016). Higher incidence is linked to social determinants of health, including higher poverty, lower insurance coverage, and stigma (Adimora, Ramirez, et al., 2014; CDC, 2016; Pence et al., 2007). HIV incidence in the South excessively affects African Americans (CDC, 2016), a likely consequence of historical and current racism, medical mistrust, and higher incarceration rates of African Americans than of other populations (Adimora et al., 2009; Adimora, Ramirez, et al., 2014; CDC, 2017; Cené et al., 2011; Earnshaw et al., 2013; LaVeist et al., 2009).

African American women experience higher HIV risk compared with other U.S. women. Comprising approximately 12% of the U.S. female population, African American women make up 61% of new HIV cases, 16 times the rate of European American women (Hodder et al., 2013). Among African American women, those living in regions with high poverty rates have five times the incidence of HIV compared with the general population of African American women. The greater prevalence of HIV among African American women is not due to higher rates of unprotected sex but rather to systemic factors (Adimora et al., 2006). A large study of women in the eastern United States showed that multilevel syndemic factors, including but not limited to poverty prevalence, discrimination, gender imbalances, community violence, and housing challenges, contributed to women’s vulnerability to HIV in the United States (Frew et al., 2016). For example, a higher occurrence of concurrent sexual relationships in African American communities, which enhances transmission of sexually transmitted diseases including HIV (Cené et al., 2011; Hodder et al., 2010; Newsome & Airhihenbuwa, 2015), is attributed to a lower proportion of men than women among African Americans, linked to African American men’s elevated rates of homicide and mass incarceration (Adimora & Auerbach, 2010; Davis & Tucker-Brown, 2013). With fewer men in their communities, African American women may acquiesce to tolerate conditions in their relationships, such as partners having multiple relationships which can increase their risk of sexually transmitted infections (Adimora et al., 2009; Dean & Fenton, 2010). Systemic factors have also been linked to participation in transactional sex, which places women at elevated risk. For example, in a study in North Carolina, increased transactional sex was associated with food insecurity, housing instability, and partner incarceration (Stoner et al., 2019).

The abovementioned social and structural risk factors are important when considering the intersectional identities of African American women living in public housing in the southeastern United States (Crenshaw, 1991). Although studies have demonstrated a link between structural factors and African American women’s elevated HIV risk, few studies have assessed women’s perspectives on the factors that women perceive to place them at increased risk of HIV (CDC, 2013, 2015; Gupta et al., 2008). Understanding these phenomena from the perspectives of African American women may shed new light on their mechanisms of action and suggest appropriate means of intervention. This study sought to understand the views of African American women living in low-income neighborhoods regarding factors they believe put African American women at risk of HIV, including use of HIV prevention services and personal sexual health behaviors. This study is the formative phase of a pilot study, seeking to adapt (an ultimately feasibility test) for African American women living in public housing communities in a small urban city in the southeastern United States, an existing CDC evidence-based intervention, the Real Aids Prevention Project-High Impact Prevention (RAPP-HIP; Lauby et al., 2000). RAPP-HIP is a community mobilization program designed to reduce HIV risk among women in high-risk communities by increasing condom use. The program includes five core components (peer network outreach, role modeling, small group activities, stage-based one-on-one encounters, and community network outreach), each of which can be used together or individually. We sought to adapt three of the components (small group activities, peer network, and community network outreach) for our public housing communities. More detailed description of the RAPP-HIP intervention is available from the CDC at https://www.cdc.gov/hiv/research/interventionresearch/rep/packages/rapp.html. This qualitative analysis contributes important perspectives from the pilot study’s target community member into the overall intervention planning, considering structural barriers and community strengths for improved HIV infection prevention within this disproportionately affected population.

## Method

### Overview of Study Design

We conducted seven focus groups among women living in the 10 low-income housing communities that comprised the majority of the local housing authority’s residents in one southeastern urban city in North Carolina. The focus groups aimed to contextualize barriers to implementation of RAPP-HIP and other HIV prevention services for southern low-income communities. The study was developed and conducted by a Community–Academic Partnership (CAP) composed of several residents and two staff members from the local public housing authority, community-based organization staff, other community stakeholders interested in HIV prevention, as well as researchers, staff, and students from local universities. The CAP was convened using snowball sampling beginning with our colleagues from HIV prevention community-based organizations and from the city Housing Authority. Individuals were invited to join and attend monthly meetings for which they were compensated at a rate of $15.00 per hour of meeting time. The CAP engaged in all aspects of the research: focus group guide development, community selection, recruitment, and interpretation of findings.

The CDC recommended conducting focus groups with the local population to adapt RAPP-HIP to local circumstances before implementation. The focus group guide used the CDC RAPP-HIP training manual discussion guide and adapted the wording with input from our CAP regarding content and phrasing (CDC, 2020). Our guide was also informed by the Gelberg–Andersen Behavioral Model for Vulnerable Populations of Healthcare Utilization which postulates that relationships between the environment (health systems and the external environment) and population characteristics predict the use of health programming, such as HIV prevention services, and personal health choices, such as HIV prevention behaviors, in turn affecting risk (Gelberg et al., 2000). Population characteristics include factors that predispose, enable, and reflect the need (evaluated and perceived) of individuals to make health-related decisions. The final focus group guide sought to contextualize environmental and population-level barriers to accessing HIV prevention programs and following safer sex practices for women in low-income housing communities (Justman et al., 2015).

All study procedures were approved by an Institutional Review Board for Human Subjects Research and all focus group participants provided written informed consent.

### Study Sample and Recruitment

We recruited a convenience sample. Eligible participants self-identified as African American women 18 years of age or older living at one of 10 public housing developments in one small southeastern city. Individual HIV status was irrelevant to recruitment criteria and not collected. Study staff posted recruitment flyers in public housing community centers and handed them out at community events to invite women to participate. Focus groups were conducted in community centers within the public housing communities. CAP members who were residents of the housing communities at times provided referrals of potential participants they knew had interest who were then screened for eligibility by study staff. Between three and 12 women participated in each focus group discussion (48 women total).

### Data Collection

Focus group discussions were held in housing authority community centers. Each focus group was conducted by a moderator, co-moderator, and note-taker, audio-recorded, and transcribed verbatim by a professional transcription service. Moderators and co-moderators were African American women from the community with bachelor’s or master’s degrees in public health and training and previous experience in conducting focus groups. We elected to conduct focus groups both because they were recommended by the CDC’s RAPP-HIP program manual and we were interested in gaining a community perspective on topics that would arise from group conversations (CDC, 2020).

### Data Analysis

**Figure 1.**
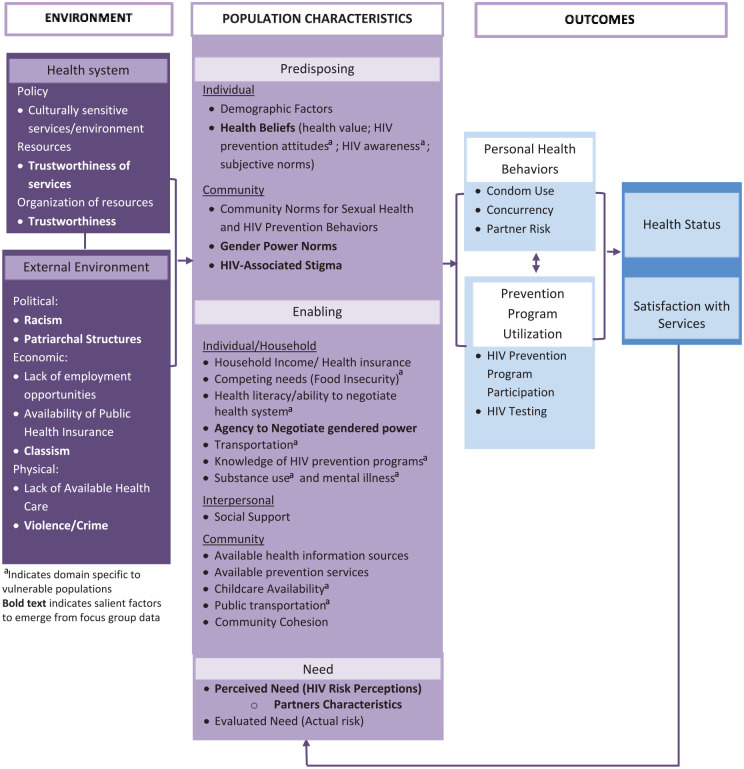
Gelberg–Andersen behavioral model for vulnerable populations adapted for HIV prevention among women living in low-income housing. *Note.* Bold text indicates salient factors to emerge from focus group data. ^a^Indicates domain specific to vulnerable populations.

We used Atlas.ti 8 to manage the transcribed data during analysis. A team of three graduate students in public health with qualitative research and analysis training, supervised by a faculty member and project manager, reviewed the transcripts and created a preliminary codebook of topical and interpretive codes derived from common words, statements, and themes in the transcripts. Topical codes were applied to words and phrases related to topics queried from the discussion guide. Interpretive codes were applied to ideas expressed but not explicitly queried or stated (i.e., trust, motivation). Intercoder reliability measures ensured consistency among the three coders, involving review for agreement in code application to multiple passages from the transcripts. Researchers used the final codebook to code the transcripts and examined codes and quotations for clusters of meaning related to contextual factors influencing women’s use of HIV prevention practices and programs, and identified points of overlap and contrast within and across each discussion. Findings from the analysis were organized into the Gelberg–Andersen model (Figure 1) to understand factors that affect HIV prevention services and practices used among the study population. The Theory of Gender and Power was used to inform our analysis of the women’s experiences with their sexual partners (Rinehart et al., 2018; Wingood & DiClemente, 2000; Wingood et al., 2009).

## Results

Participants were all cis-gender African American heterosexual women with annual household incomes of less than $20,000 whose ages ranged from 24 to 81. Findings presented below are organized by the conceptual pathways of the final adapted Gelberg–Andersen model (Figure 1; Becker, 1974; Young, 1992). The findings are presented in the following order: (1) a theme related to effects of the health care system: *perceptions of health care systems as untrustworthy*; (2) themes related to the external environment, termed *structural oppression* and includes four subcategories; (3) findings related to population characteristics: *predisposing factors* (i.e., *health beliefs, community-level HIV-associated stigma*), *enabling characteristics* (i.e., *agency to negotiate gendered power, community cohesion, trust in health care systems*), and *need factors* (i.e., *perceived HIV prevention needs, perceived partners’ characteristics*).

### Perceptions of Health Care Systems as Untrustworthy

Participants asserted health care providers do not have their best interests at heart. Participants believed health care providers gave false, insufficient, or detrimental health information to women from their communities based on personal experiences, leading to negative perceptions of service providers and distrust of health care systems more generally.

Participant A: I went to [hospital], they said, “Ain’t nothin’ wrong with you.” I left, they discharged me . . . I was still hurting on the same side, on my side . . . . I went back on the same night, 3:00 in the morning, I drove myself. Mm-hmm. They said I made it there just in time. I don’t know why y’all didn’t see this when I was here earlier . . . . They wasn’t doing their job . . . . Kidney—my kidneys failed on me. Yeah. That’s why . . . I don’t like their hospital.

Participant B: They’ll see you, get a little money, and then they send you on about your way, and I feel like that’s not right.

While most participants expressed a distrust of health care providers, a few women perceived their providers as caring and acting in their best interests.

She [doctor] seems more like my sister or my cousin or something. We’re so close that I tell her everything. And seems just more like my sister. We’ve got a good relationship. And she’ll call me, and she’ll say—tell me about the swab tests, what would help—. . . She say, “You’re doing fine.”

Across focus group discussions participants expressed a belief that they best received health information from individuals who were relatable and familiar to them.

### External Environment: Structural Oppression

Women raised concerns about broad societal factors that negatively affected them, factors which reflected structural oppression, injustices applied to specific groups by societal structures, such as bureaucratic hierarchies, market mechanisms, and oppressive and stereotyping beliefs and norms (Adimora, Hughes, et al., 2014). Forms of structural oppression participants discussed having affected their use of health behaviors and services related to HIV prevention included *racism, discrimination based on social class, unequal gender power dynamics*, and *neighborhood violence and crime*. In some cases, women articulated structural oppression as a root cause of accessibility to HIV prevention services, whereas in others, the researchers have inferred them from women’s descriptions of their experiences. At times, multiple forms of structural oppression were found to intersect (Crenshaw, 1991).

### Racism

Participants discussed experiences of racial discrimination and disadvantage, particularly regarding the quality of health communication and services they received. Participants perceived themselves within broader power structures, where knowledge and resources were created and held by European American people to oppress communities of color.

My grandson who I’m raising, I’m telling him yesterday that—we were talking about slavery. [. . .] He was like, “Aren’t you glad you’re not in slavery? That African American men are not enslaved?” And I said, “We are in a way, in a sense. What defines slavery for you? Because slavery is anything you enslaved to.” We’re enslaved to drugs. We’re enslaved to a lot of things. Poverty. A lot of things.

Many of the examples given by participants included experiences of providers’ implicit racial biases. In some cases, participants’ perceived failures of health professionals to communicate in a culturally sensitive manner made women feel mistrustful. This mistrust prevented women from understanding the effects personal health behaviors can have on health outcomes.

I don’t wanna say it like this, but even when we go to the doctor, the white people’s terminology, “Eat healthy, don’t smoke, don’t do drugs, protect your sex or abstinence.” [. . .] I smoked cigarettes the whole nine months. I even smoked a little weed the whole nine months. [. . .] And I drank some beer the whole nine months. My baby—my last baby was 9 pounds, even. [. . .] So I don’t know what he’s talking about.

Participants perceived that implicit racial biases of health service providers led to inadequate communication of health information to African American women. Women questioned medical recommendations they perceived to reflect racial bias:

They [healthcare providers] don’t communicate. “Hey, we’re going to give you this shot. It’s birth control. Here you go.” But see, us black people have to start being more aware ourselves. We got to start asking questions. You can’t just stick me in my arm with anything no more.

### Discrimination Based on Social Class

Women perceived that classism and the institutionalized profit motive within much of the U.S. health care system interfered with equitable provisions of health services, adding to existing mistrust. Participants perceived they had experienced both undertreatment and overtreatment due to the provider’s implicit racial and class biases and financial incentives:

Some doctors won’t see you at all, like, especially when you don’t have no insurance, they will not see you at all.

——————————-

I went to [health center] one day. They said, “Oh, you might have an STD. So what we’re going to do is we’re going to treat you.” And I came back with no STD. So you’re not treating me. First of all, you falsely medicating me [. . .] You’re taking insurance money that don’t need to be used. [. . .] I think because they think we’re black and ignorant and they can tell us anything and we going to fly with it.

### Unequal Gender Power Dynamics

**Figure 2.**
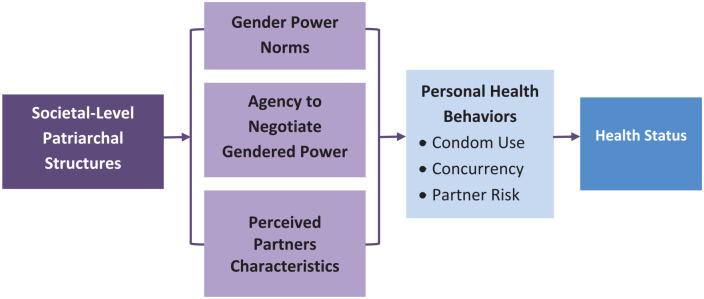
Hypothesized relationships among the environmental characteristics (patriarchal structures) and three population characteristics (gender–power norms, agency to negotiate gendered power, and perceived partner characteristics) and the effect on health behaviors.

Participants spoke of challenges pertaining to existing gender-based power imbalances in their communities influencing women’s abilities to negotiate safer sex [Figure 2]. These included male sexual partners having concurrent partners and/or failing to disclose their non-monogamy and/or HIV status to partners. The quote below illustrates women’s mistrust of sexual partners:

I like him, [. . .] I don’t trust him, and I go to the doctor behind his back and stuff. And I tell him, “I’m going to the doctor. I figure something’s wrong with me. You’ve got to go, man. You’re going in.” Now a man is sick, he don’t tell you. Now how you going to find out?

Within this imbalanced power structure, men’s needs as well as the need to have a man are often seen as paramount:

Participant A: “[The mother will] take that child’s plate and eat it, and share it with their boyfriend and the child is still at square one. Because the person took the food from [the child] and ate it and gave it to the boyfriend. [. . .]”

Participant B: “Because they don’t want to do without a man. So they’re looking for somebody and they settle for anybody and it turns out to be a nobody.”

Moderator: “What does that say about the need for HIV prevention?”

Participant B: “A lot.”

The discussion then links back to men’s failure to disclose concurrency of sexual partners and women’s lack of trust in their boyfriends as connected to HIV/STI risk.

Participant: “Now if you’re having sex with a man you’ve got to protect your body because simply you don’t know whether he’s sick or not: he won’t tell you. Why they won’t tell you? That’s sick. They will not tell you.”

Moderator: “Even if you ask?”

Participant: “You can meet a good-looking man, you think—and I’m single, you know, and I’d sort of like to have a little fun, and you be thinking that—you could talk to him—you be wanting to talk to him, you don’t know whether he’s sick or not. They used to would tell you a long time ago.”

### Neighborhood Violence and Crime

Another aspect of the external environment that concerned women was the level of violence—family, gang, and gun—in their communities. Such violence created mistrust within the community, threatened children’s safety, and caused residents to miss out on services and resources. Participants perceived HIV prevention-related agencies ceased coming to the communities due to personal safety concerns.

Participant A: “I mean this is just the beginning of the year –”

Participant B: “We’ve had over 60 murders already.”

Participant A: “Yeah. Like I don’t even go to funerals anymore. But if I did go to every funeral this year already I would have already been at 13 funerals. And it’s just the last day of February. That’s very sad for a community that—we have the potential of being so much more.”

——————————-

I just feel like most of them [health services] don’t come around like that no more. Because like what y’all said, like, y’all should feel comfortable and safe coming out here trying to help people that need the help, but the riff-raff is just gonna show y’all that y’all not safe out here—why would y’all come?

### Population Characteristics: Predisposing Factors

#### Health Beliefs

Participants viewed HIV as a critical health issue for African American communities:

Well I’m speaking from a personal reference, my father died. I watched my father suffer, from walking normal one day to turning into a complete vegetable. AIDS is a big epidemic in our black community.

Despite the belief that HIV is an important issue within their communities, participants perceived community members lacked an understanding of HIV. Misperceptions included beliefs that HIV could be ruled out among individuals with a certain aesthetic or that it occurred among certain groups, such as the lesbian, gay, bisexual, transgender, and queer or questioning (LGBTQ) community.

A lot of people out here thinking because they’re real skinny that they are healthy. I tell them all. No, that’s not it.

——————————-

You can be sitting on your porch, and you’ll see a whole bunch of gay boys and girls hanging in groups. So, I’m sitting there thinking, “I wonder have they been tested for AIDS or HIV,” because some womens go both ways. Some mens go both way, or even both.

#### Community-Level Stigmatizing Attitudes Toward HIV

Participants talked about how stigma toward HIV kept people from accessing HIV treatment and prevention services in their community.

Do I want to go in the clinic and everybody know that’s an HIV clinic? . . . No, because when I’m walking out that door everybody from my community see me walking out that door—somebody in my community walks in there and they go home, they’re gonna tell, like, “Girl, you know such-and-such was in the HIV clinic.”

### Population Characteristics: Enabling Factors

#### Agency to Negotiate Gendered Power

**Figure 3.**
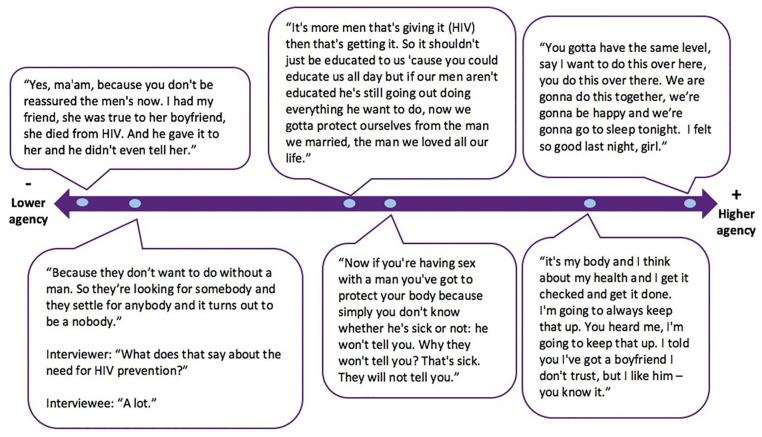
Spectrum of women’s agency to negotiate gendered power.

Women’s descriptions of their agency to negotiate gendered power and norms in their sexual relationships, and its affect on their safer sex practices and use of preventive care, differed. Figure 3 depicts illustrative quotes reflecting the range of experiences women reported, from powerlessness, to insistence on being “on the same level” with their sexual partner.

#### Community Cohesion

All participants expressed a desire for cohesion, trust, and belonging within their community. Some felt strong ties with their neighbors, expressing an obligation to take care of their neighbor, and pride in their community. A sense of belonging contributed to greater perceived collective resilience toward health challenges and enabled positive health behavior.

I have seen now in [public housing community], especially where I live and people that I know that something that you need or if there’s a resource, I think that we relate it to one another. [. . .] the majority, if something happens, everybody gather around together. If you make your need known [. . .] the people in this community are like family.

Those who saw their communities as noncohesive linked these views to experiences of violence, and a lack of safety and resources. Participants suggested community unity was a means to countering external environmental factors inhibiting health services access.

If our young men had more to do on these streets, they wouldn’t be out here shooting and killing each other every day, but they can’t. So it really starts in the community; and I feel like if we changed our community, we change ourselves, then we won’t have so much negativity in our community.

Across discussions, women expressed that their identities as mothers and caretakers made them responsible for fostering community cohesion. Men were also viewed to have important roles to play.

Instead of us getting together and pulling together and standing strong with each other, we’re starting to fall apart. So I feel like as us being parents and mothers [. . .]—as us being women and we’re strong the way we are, we should start pulling together more instead of pulling apart.

——————————-

It’s not only women—like male roles are very important and we don’t get those enough and that’s what really tears down our community even more because the male roles aren’t playing male roles.

### Other Enabling and Hindering Factors

The biggest hindrance to the use of health care services was a lack of financial resources including the lack of health insurance. Residents described difficulty accessing services located away from their community, even short distances, due to their inability to afford a car, have access to a ride, or pay for public transportation. Inability to pay for childcare was another barrier commonly cited. The following quotations illustrate how lack of resources hindered access:

I got friends, girl, that can’t even get a mammogram because they don’t have the insurance—they ain’t had mammograms in 15 years. I had one friend had breast cancer, and she died. She didn’t have insurance to get a mammogram.

——————————-

Because this is a big community and there’s a lot of women out here and a lot of women are here because we have a lot of kids so it’s kind of hard for us to get babysitters and transportation and stuff like that for our kids and for ourself.

### Population Characteristics: Perceived Need

Despite the lack of trust in the health care system, participants felt that the community needed and desired health programming, particularly sexual health education programming:

I think [health programming] needs to be out here because we have a lot of families that are infected, and may not know about it. It’s a lot of guys who have multiple households; guys who know they are infected with STDs. So there needs to be a program out here.

——————————-

It needs to be known and studied. People need to be more aware of what HIV is, where it comes from, how to protect yourself from it. It’s—my children—I ask my children—they don’t even know what it is. Yeah, it’s an issue that’s hidden that really needs to be spread worldwide.

## Discussion

African American women living in low-income housing communities in a small southern city identified HIV risk as an issue of serious concern in their communities. Participants expressed a desire to have HIV prevention interventions available to their communities. Women were highly aware of the myriad societal-level phenomena that contribute to health disparities, including HIV risk. These findings provide insight into the effects of such phenomena on the daily lives and health of individuals. Women’s views in this study were consistent with scientific findings from studies demonstrating a link between structural factors and African American women’s risk of HIV infection (Adimora et al., 2006; Blackstock et al., 2015; Frew et al., 2016; Stoner et al., 2019; Tims-Cook, 2019; Valdiserri & Holtgrave, 2019; Williams et al., 2010). Blackstock et al. (2015) found that women living in low-income communities perceived their communities to be at elevated HIV/STI risk, mostly due to contextual and structural factors such as access to health care and education. Our findings, with those of others, present a compelling case for the need to situate HIV prevention efforts within an intersectional framework recognizing the effects of these compounding and interconnected structures. The insights provided by participants further indicate the value of including women from communities targeted for HIV prevention interventions as important partners in intervention development and evaluation (Carpenter et al., 2009).

Women perceived structural oppression to occur within their communities and inside health care systems. These influences in conjunction with financial barriers appeared to affect women’s use of sexual health services. Women were skeptical of medical advice and distrusted that the health care providers would act in their best interest. Greater medical mistrust among African Americans demonstrated elsewhere has been associated with lower rates of using prevention services (Armstrong et al., 2008; Armstrong et al., 2013; Halbert et al., 2006; Musa et al., 2009; Nguyen et al., 2009; Patel, 2017; Voils et al., 2005). In our study, women’s distrust stemmed from personal experiences of racism and classism within health care, resulting in decreased access to health services. A national probability sample study showed that racial differences in health care system distrust were fully mediated through African American individuals’ previous experiences of racial discrimination (Tekeste et al., 2019).

Examples of service providers successfully establishing open and respectful relationships with public housing residents also came through in the data, albeit less commonly. This suggests that, despite historical events and personal experiences that provide African American women reasons to distrust the health care system, trust between clinicians and patients can be established at the individual level through providers’ demonstrated commitments to and genuine engagement with low-income housing communities. Sexual health programs that increase investment in trust and relationship building through community engagement may be more likely to succeed. In reacting to descriptions of the RAPP-HIP program, participants did say they thought that they would feel comfortable receiving information in larger group educational sessions and small discussion groups with other women.

Women commonly discussed worry that male partners’ sexual behavior may put them at an unknown risk of HIV. While we are unsure whether women’s perceptions of their partners were accurate, they affect behavior and choices. Women felt frustrated with their lack of control over men’s potentially risky behaviors and, therefore, their ability to protect themselves. Gendered power dynamics further hindered women’s capacities to make positive health behavior choices. The Theory of Gender and Power has been shown to help frame gendered relationship dynamics, such as those identified here (Hill et al., 2017; Rinehart et al., 2018; Wingood & DiClemente, 2000; Wingood et al., 2009). Our findings reiterate other studies showing that women who face gender ratio imbalance may lack negotiating power in relationships with men, tolerate less preferred, less economically stable or nonmonogamus partners, and acquiesce to unprotected sex or concurrent sexual relationships (Bowleg et al., 2004; Newsome & Airhihenbuwa, 2015).

Male sexual partners of participants in this study are likely to have experienced the same societal conditions affecting women, including racism, classism, and unjust policies (Adimora et al., 2006; Aral et al., 2008; Harris et al., 2010; Wingood, 2003). Participants suggested engaging men in programs aiming to prevent HIV among women is essential to holistic community-level interventions.

While the findings of this study reveal challenges faced by African American women living in public housing residences, participants displayed determination to strengthen community cohesion and improve peers’ health. Leveraging the existing knowledge base regarding HIV prevention and addressing misperceptions is key to successful implementation of sexual health programs.

This study had limitations. The perspectives reported here were from women living in public housing communities in one southeastern city and may not reflect the experiences of other African American women in low-income communities. Moreover, our sample was a convenience sample and while we generally did not encounter major roadblocks to recruitment, for 4 of the focus groups, at least one scheduled potential participant did not show up to participate. It is likely that those who chose to schedule and show up to the focus groups are not representative of all women living in the housing communities. While the goal of this qualitative study was not to assess prevalence of phenomena, it is possible we missed themes that may have emerged if other individuals had participated. Participants may have provided socially desirable responses, as women in each focus group were members of each others’ communities. Recall bias in their responses is possible, given the varied time between their experiences and the discussions. In addition, although we received helpful input from CAP members regarding ways to simplify the focus group guide questions’ wording, there were times when some participants seemed to struggle to fully grasp the questions being asked and discussions broadened to cover areas of all aspects of challenges that their communities faced, not just those that were health or sexual health related.

Despite these limitations, the findings offer important insights. Even with the concerns regarding distrust of medical and academic professionals, women articulated a desire for HIV prevention programs, expressing commitments to collaborative partnerships with peers and trustworthy external entities to reduce HIV risk within their communities.
